# Evidence-Based Considerations Exploring Relations between SARS-CoV-2 Pandemic and Air Pollution: Involvement of PM2.5-Mediated Up-Regulation of the Viral Receptor ACE-2

**DOI:** 10.3390/ijerph17155573

**Published:** 2020-08-02

**Authors:** Marina Borro, Paolo Di Girolamo, Giovanna Gentile, Ottavia De Luca, Robert Preissner, Adriano Marcolongo, Stefano Ferracuti, Maurizio Simmaco

**Affiliations:** 1 Laboratory of Clinical Biochemistry, University Hospital Sant’Andrea, Department of Neurosciences, Mental Health and Sensory Organs, Faculty of Medicine and Psychology, Sapienza University, via di Grottarossa 1035, 00189 Rome, Italy; marina.borro@uniroma1.it (M.B.); giovanna.gentile@uniroma1.it (G.G.); 2 School of Engineering, University of Basilicata, viale dell’Ateneo Lucano 10, 85100 Potenza, Italy; paolo.digirolamo@unibas.it; 3 Laboratory of Clinical Biochemistry, University Hospital Sant’Andrea, via di Grottarossa 1035, 00189 Rome, Italy; ottavia_deluca@yahoo.it; 4 Structural Bioinformatics Group, Charité–Universitätsmedizin Berlin, Philippstr. 12, 10115 Berlin, Germany; robert.preissner@googlemail.com; 5 General Direction, University Hospital Sant’Andrea, via di Grottarossa 1035, 00189 Rome, Italy; amarcolongo@ospedalesantandrea.it; 6 Department of Human Neuroscience, Sapienza University, Piazzale Aldo Moro, 5, 00185 Rome, Italy; stefano.ferracuti@uniroma1.it

**Keywords:** COVID-19, SARS-CoV-2, PM_2.5_, angiotensin-converting enzyme 2, xenobiotic response element, aryl hydrocarbon receptor

## Abstract

The COVID-19/SARS-CoV-2 pandemic struck health, social and economic systems worldwide, and represents an open challenge for scientists —coping with the high inter-individual variability of COVID-19, and for policy makers —coping with the responsibility to understand environmental factors affecting its severity across different geographical areas. Air pollution has been warned of as a modifiable factor contributing to differential SARS-CoV-2 spread but the biological mechanisms underlying the phenomenon are still unknown. Air quality and COVID-19 epidemiological data from 110 Italian provinces were studied by correlation analysis, to evaluate the association between particulate matter (PM)_2.5_ concentrations and incidence, mortality rate and case fatality risk of COVID-19 in the period 20 February–31 March 2020. Bioinformatic analysis of the DNA sequence encoding the SARS-CoV-2 cell receptor angiotensin-converting enzyme 2 (ACE-2) was performed to identify consensus motifs for transcription factors mediating cellular response to pollutant insult. Positive correlations between PM_2.5_ levels and the incidence (r = 0.67, *p* < 0.0001), the mortality rate (r = 0.65, *p* < 0.0001) and the case fatality rate (r = 0.7, *p* < 0.0001) of COVID-19 were found. The bioinformatic analysis of the ACE-2 gene identified nine putative consensus motifs for the aryl hydrocarbon receptor (AHR). Our results confirm the supposed link between air pollution and the rate and outcome of SARS-CoV-2 infection and support the hypothesis that pollution-induced over-expression of ACE-2 on human airways may favor SARS-CoV-2 infectivity.

## 1. Introduction

Northern Italy was among the first and worst SARS-CoV-2 affected regions in Europe. After about four months from the identification of the primary infection, the Lombardia Region still counts for about half of the total cases, new infections and total deaths. The peculiarity of the pandemic spread in a relatively small part of the country may represent a milestone to dissect and assess single factors contributing to the exacerbation of the pandemic. Italy is a small, densely populated, highly interconnected country. Industrial production and per capita wealth are distributed from north to south following a descending trend [1]. Thus, the high human mobility (national and international) in North Italy, which represents the industrial and business core of Italy, was suggested as the primary catalyzing factor for the SARS-CoV-2 spreading [2]. Yet, this is just a part of the story since other Italian regions, as Veneto, bordering on Lombardia, with a similar economic and socio-demographic background, or Lazio (Central Italy, where Rome is located) with a similar exposition to human mobility, were much less hurt. Regional differences in the health management system have also been hypothesized as additional factors affecting the intensity of the pandemic [3], especially in the early phase, before the onset of strict containment policies by the National government. The third macroscopic factor markedly different between Lombardia and the rest of Italy is the level of air pollution. Since the relationships between air pollution and different respiratory diseases are widely recognized [4,5,6], a causative role in the SARS-CoV-2 pandemic has also been hypothesized. To test the hypothesis and to estimate the extent to which exposition to air pollution can increase the risk of viral infections, we performed a statistical analysis correlating the number of infected people and COVID-19 mortality indexes in the period 20 February–31 March 2020 to the particulate matter (PM)_2.5_ concentration levels in 110 Italian provinces in the period 16–26 February 2020. The analysis categorized in terms of provinces allowed better appreciation of the effects of differential air pollution levels on viral infection rate and severity.

We suggest a possible biological connection between PM_2.5_ exposure and the human SARS-CoV-2 receptor protein ACE-2 (angiotensin-converting enzyme 2), supported by a bioinformatic analysis of the ACE-2 gene promoter region. PM_2.5_ particles contain different molecules, including polycyclic aromatic hydrocarbons (PAHs), heavy metals and volatile compounds [7,8,9]. PAHs are known to stimulate eukaryotic cell response by activating the transcription factor aryl hydrocarbon receptor (AhR), which in turn activates the expression of target genes by the binding of specific DNA consensus motifs, the xenobiotic response element (XRE) and the non-canonical xenobiotic response element (NC-XRE) [10,11,12,13]. We identified such motifs in the regulative region of the ACE-2 gene.

## 2. Materials and Methods

### 2.1. Data Sources and Statistics

The effective impact of PM_2.5_ particles on COVID-19 infection outbreak is expected to be strongly dependent on particles persistency in the air, i.e., on the duration of the effective exposure of the human respiratory system throughout the weeks preceding the pandemic onset. Thus, considering a possible time gap between population exposure to enhanced PM_2.5_ concentration levels and the onset of the infection and the eventual death of patients, we carried out a study to correlate the number and outcome (e.g., mortality) of COVID-19 cases in the period 20 February–31 March 2020 versus the corresponding average PM_2.5_ concentration values observed in the period 15–26 February 2020.

**Figure 1:**
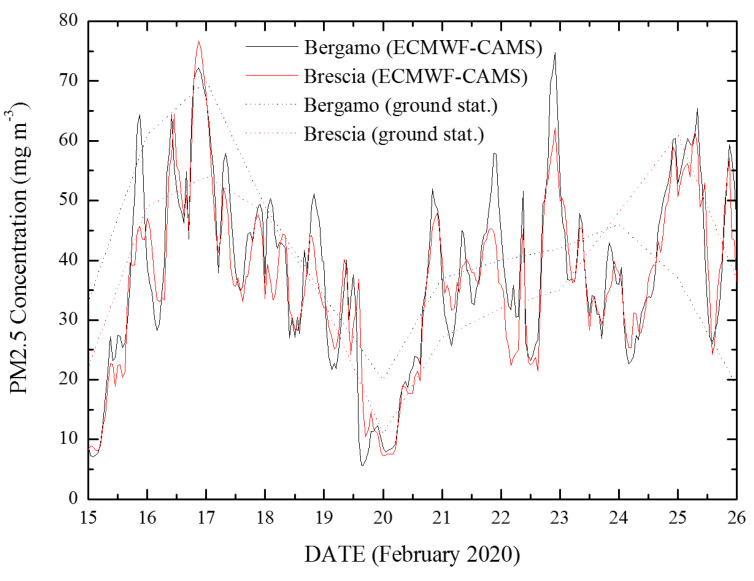
Particulate matter (PM_2.5_) concentration levels over the period 15–26 February 2020 as measured by the two ground stations in Bergamo (via Meucci, 45°41′24′′ N, 09°38′28′′ E) and Brescia (Villaggio Sereno, 45°31′04′′ N, 10°10′41′′ E), together with the data from near-real-time European Centre for Medium-Range Weather Forecasts (ECMWF)–Copernicus Atmosphere Monitoring Service (CAMS) analysis.

PM_2.5_ concentration measurements at ground level were carried out by one air quality monitoring station for each province. The ground-based network of air quality monitoring stations is run by the Regional and Provincial Environment Agencies, which manage air quality monitoring networks on behalf of regional and national governments. For those provinces for which PM_2.5_ concentration measurements from ground stations were not available, corresponding analyses produced by the Copernicus Atmosphere Monitoring Service (CAMS) of the European Centre for Medium-Range Weather Forecasts (ECMWF) were used, since these PM_2.5_ values reproduce well measurements from ground stations (Figure 1). ECMWF–CAMS analyses are provided with hourly resolution and a grid size of 10 × 10 km. The CAMS near-real-time reanalysis is the most recent global reanalysis data set of atmospheric composition and air quality [14], with a demonstrated unprecedented level of accuracy and space–time resolution.

Data on the population for all Italian provinces has been extracted from the ISTAT Dataset [15], while the number of infected patients has been extracted from the Italian Civil Protection Department database [16]. The number of deaths due to COVID-19 over the time period 20 February–31 March 2020 for the 110 Italian provinces are from the Italian Statistics Institute (ISTAT) report [1].

Data include 107 provinces as four provinces in Southern Sardinia (Carbonia-Iglesias, Ogliastra, Olbia-Tempio and Medio Campidano) have been grouped together as “Sud Sardegna”.

The “incidence of the pathology”, which quantifies the frequency with which a pathology appears in a particular population [17], was defined as the number of infected people in a province over the province population (infected/population ratio). The “mortality rate” for COVID-19 was defined as the number of deaths normalized to the province population [18], which quantifies the frequency of death occurrence in a defined population. The “case fatality rate” (CFR) [19], also called case fatality risk or case fatality ratio, is defined as the proportion of people who die from a specified pathology among all individuals diagnosed with the pathology over a certain period of time.

To study correlations between the above-mentioned indexes and the level of PM_2.5_, a linear fit was applied to the data using a linear regression function. Assuming that population exposure to enhanced PM_2.5_ concentration levels should precede enhanced susceptibility to viral infection, we sought to correlate number and outcome (e.g., mortality) of COVID-19 cases in the period 20 February–31 March 2020 versus the corresponding average PM_2.5_ concentration values observed in the period 15–26 February 2020. Statistics were performed using the statistical software Origin (v7.0220) by OriginLab Corporation.

### 2.2. Bioinformatic Analysis of the ACE-2 Gene

The regulative DNA sequence (promoter region) upstream of the protein coding region of the ACE-2 gene was analyzed by the BKL TRANSFAC software from Biobase Gmbh [20], which allows the identification of binding sites (e.g., consensus DNA motifs) for transcription factors. Briefly, the ACE-2 Reference Sequence (NG_012575.1) was obtained from the NCBI Nucleotide Database [21]. Five DNA kilobases spanning the promoter region were scanned to detect the XRE core element: 5′-GCGTG-3′ [12] and the NC-XRE core element 5′-GGGA-3′ [13].

## 3. Results

### 3.1. Covid-19/PM_2.5_ Correlations

**Figure 2:**
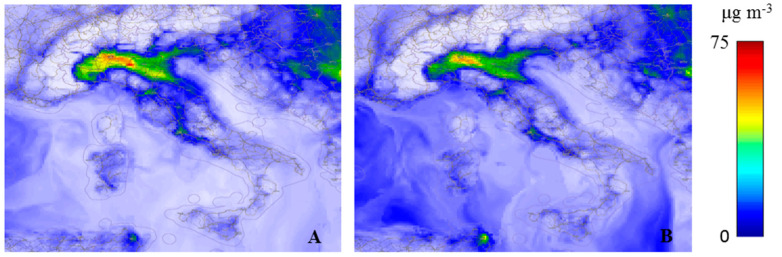
PM_2.5_ (**A**) and PM_10_ (**B**) concentration levels at 00:00 Coordinated Universal Time (UTC) on 16 February 2020 from near-real-time ECMWF–CAMS analysis over an area extending over the latitudinal interval 36–48° N and the longitudinal interval 5–20° E.

The PM_2.5_/PM_10_ concentrations in Italy, as provided by the near-real-time ECMWF-CAMS analysis few days before the identification of the “patient one”, are shown in Figure 2. Compared to the limit values of 25 µg/m^3^ for PM_2.5_ and 50 µg/m^3^ for PM_10_ stated by the WHO Air quality guideline [22], large areas in the Po Valley presented diffused and high concentrations of particulate matter, with a peak of intensity roughly corresponding with the Lombardia Region. In particular, enhanced PM concentrations were present over a large portion of the Po Valley, with levels of PM_2.5_ up to 70 µg/m^3^ and levels of PM_10_ up to 50 µg/m^3^ in Lombardia. Interestingly, while PM_10_ concentrations are typically higher than PM_2.5_ concentrations, comparable amounts of these two species were found in the last two weeks of February 2020 in most areas of the Po Valley (data not shown), with temporary situations where PM_2.5_ concentrations were found to be systematically higher than corresponding PM_10_ values by approximately 10 µg/m^3^. Figure 2 testifies to one of these occurrences. More specifically, the 12-days preceding the pandemic onset in Italy (15 to 26 February 2020) were characterized by high particle pollution, with elevated and persistent PM_2.5_ levels over large portions of the Po Valley, with an average value for the period exceeding 40 µg/m^3^. In this same period, PM_2.5_ concentration analyses in two Lombard cities, Bergamo and Brescia, revealed very high peak values (up to ~80 µg/m^3^) at specific times of the day, such peaks being not detected by daily-averaged ground station measurements (Figure 1).

**Figure 3:**
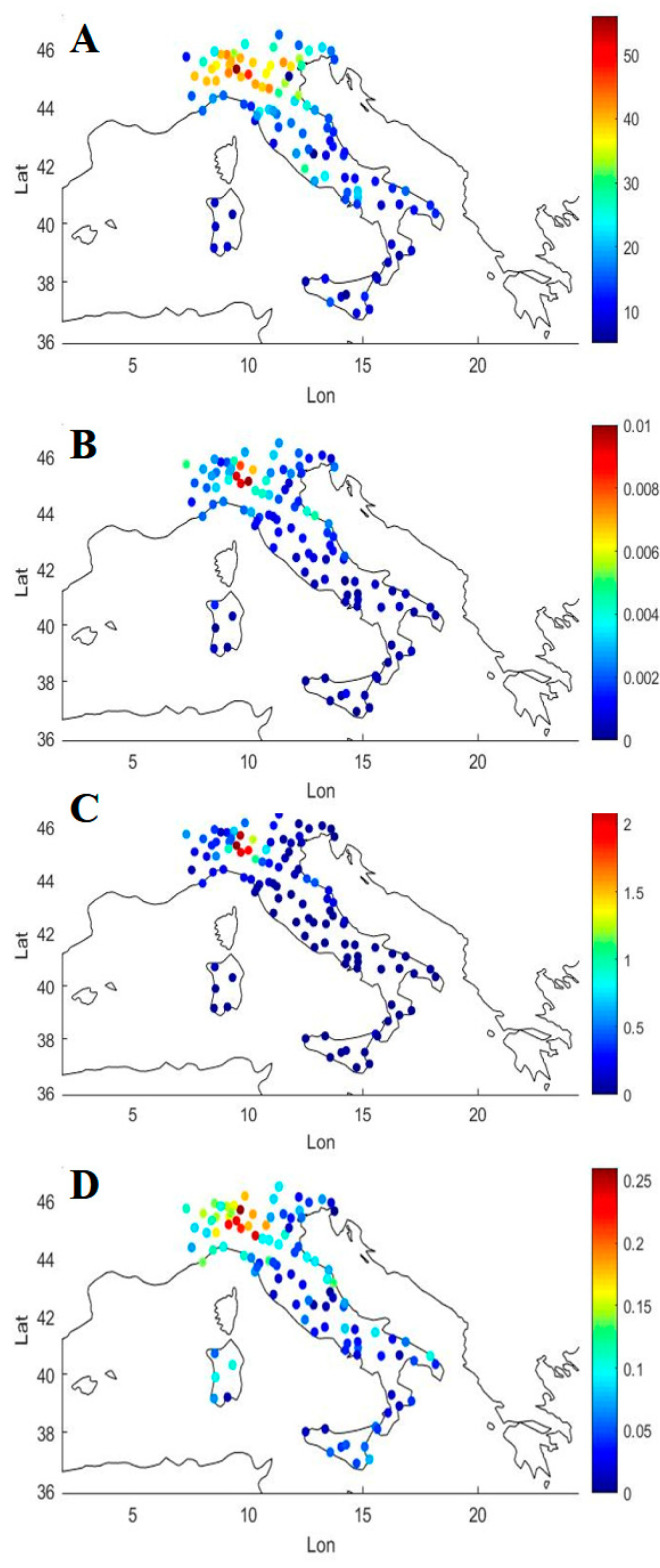
PM_2.5_ concentrations averaged over the period 15–26 February 2020 for the 110 Italian Provinces (**A**); incidence of COVID-19 (**B**), mortality rate (**C**) and case fatality risk (**D**) over the period 20 February–31 March 2020.

Surface PM_2.5_ concentration measurements from ground stations and COVID-19 epidemiology are shown in Figure 3 for the 110 Italian provinces with color-coded maps. Visual inspection reveals good overlap in the intensity scale between PM_2.5_ concentration values, averaged over the period 15–26 February 2020 (panel A), and COVID-19 epidemiology over the period 20 February–31 March 2020, e.g., the incidence of COVID-19 cases (panel B), the mortality rate (panel C) and the case fatality rate (panel D).

**Figure 4:**
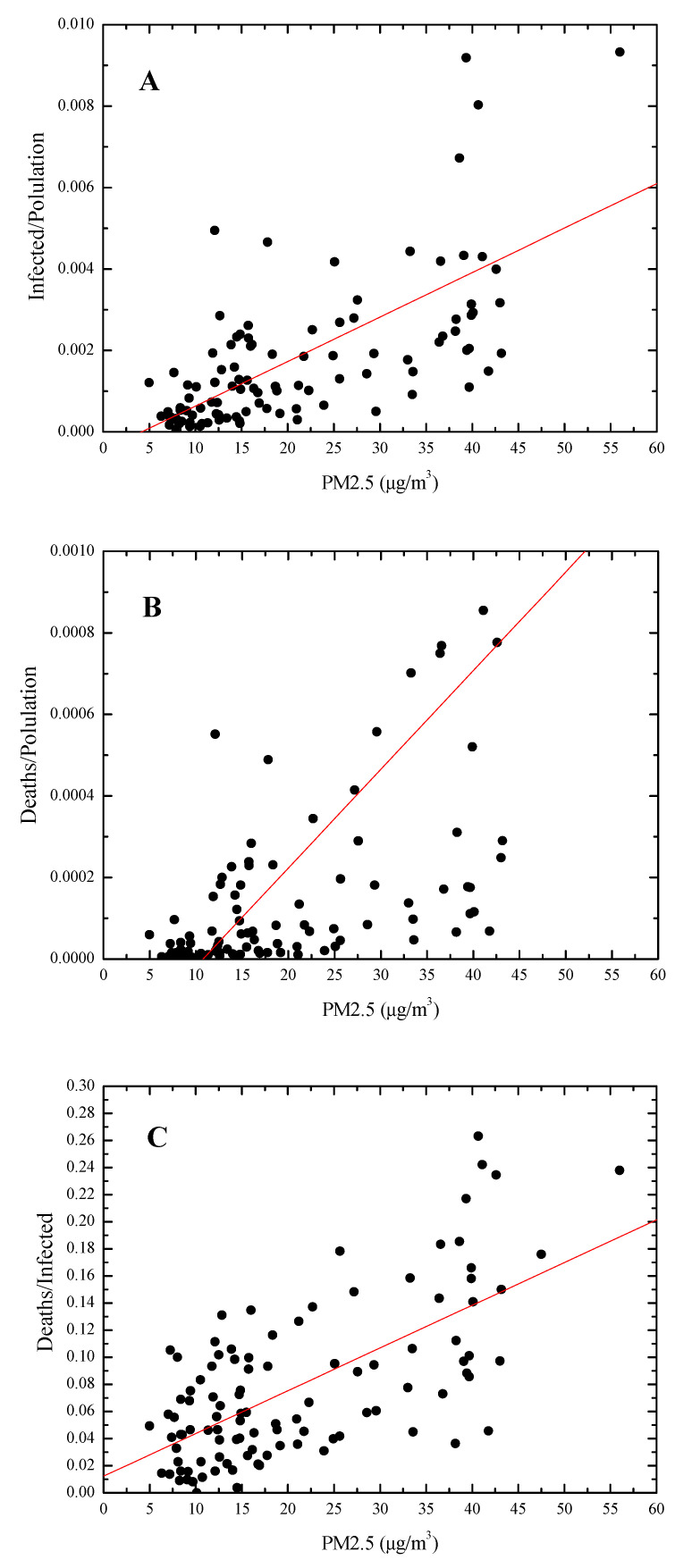
Linear regression analysis correlating the average PM_2.5_ concentration values in the period 15–26 February 2020 with the incidence of the pathology (**A**), the mortality rate (**B**) and the case fatality rate (**C**) in the period 20 February–31 March 2020.

The correlations between PM_2.5_ and COVID-19 cases and outcome were statistically significant. In detail, we found a positive correlation (r = 0.67, *p* < 0.0001) between the average PM_2.5_ level in the period 15–26 February 2020 and the incidence of COVID-19 (infected/population ratio) in the period 20 February–31 March 2020 (Figure 4, panel A). The slope of the regression line, that was (1.1 ± 0.1) × 10^-4^ m^3^/µg, implies a tripling (from 0.065 to 0.2%) of the infection rate for average PM_2.5_ concentration levels increasing from 10 to 22 µg/m^3^.

The average PM_2.5_ level was positively correlated also with the mortality rate (r = 0.65, *p* < 0.0001) and with the case fatality rate (r = 0.7, *p* < 0.0001) (Figure 4, panels B, C). The case fatality rate (deaths/infected ratio) appears to be strongly dependent on PM_2.5_ concentration, with a slope of the regression line of (3.1 ± 0.3) × 10^-3^ m^3^/µg which implies a doubling (from 4.5 to 9%) of the mortality rate of infected patients for average PM_2.5_ concentration levels increasing from 10 to 25 µg/m^3^.

None of the analyzed parameters correlated with the population density of the Italian provinces (r = 0.045, *p* = 0.65 for the infection rate; r = 0.19, *p* = 0.051 for the mortality rate, r = 0.093, *p* = 0.34 for the case fatality rate).

### 3.2. Bionformatic Analysis

**Figure 5:**
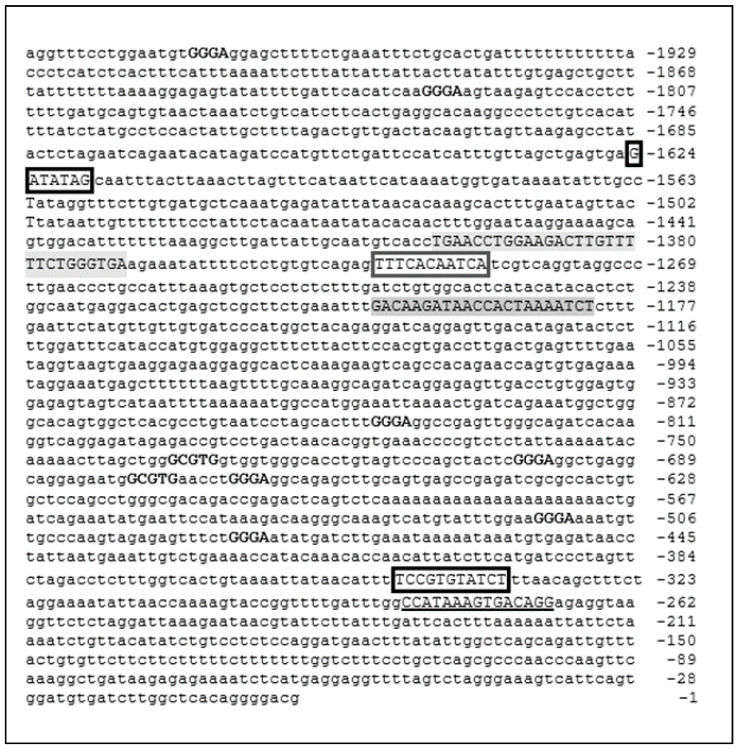
DNA sequence of the promoter region of the ACE-2 gene. Numbers indicate the position of the nucleotide upstream of the starting site for protein production. Core XRE (GCGTG) and NC-XRE (GGGA) motifs are shown in bold. Additional regulative consensus motifs are showed as follows: TATA box (underlined); GATA motifs (black boxed); STAT/FOXA/FOXO/Ets/GR/p53 motif (light grey); GATA/HNF1 motif (dark grey); C/EBP motif (grey boxed).

The DNA sequence of the ACE-2 promoter region is reported in Figure 5. Two XREs have been identified at position −673/−677 and −730/−734 and seven NC-XREs have been identified at position −481/−484, −512/−515, −663/−666, −697/−700, −833/−837, −1824/−1827 and −1960/−1963.

## 4. Discussion

In Italy, the SARS-CoV-2 pandemic strongly affected the northern part of the country, compared to other areas with similar socio–economic and demographic backgrounds or similar exposure to human movements. In the early pandemic phase, this geographical pattern could not be sufficiently explained by the containment effect of legal restrictions to human movements, which started on 9 March 2020.

North Italy is among the most air-polluted regions of the world [23]. Atmospheric concentrations of PM_2.5_ and PM_10_ are interrelated within atmospheric chemical processes involving volatile organic compounds and ozone. Primary sources of PM_2.5_/PM_10_ particles, mainly associated with biomass combustion (pellet stoves or wood), diesel engines, and vehicular mobility, are predominant in Northern Italy, but secondary aerosol formation represents an additional important source.

Enhanced levels of PM_2.5_ particles were observed over the two-month period January–February 2020 preceding the virus pandemic spread. WHO air quality guidelines for PM_2.5_ (<25 µg/m^3^) were exceeded on ~45 days over this two-month period in the most part of the Po Valley, where major effects in terms of infections and casualties occurred, with levels up to 60–70 µg/m^3^ observed within the 2–3 weeks preceding the contagious activation and with an average value in the period 15–26 February 2020 exceeding 40 µg/m^3^ over large portions of the Po Valley (data not shown).

Long-term exposure to particulate matter (PM), fine particles, and nitrogen compounds is well recognized as a risk factor for all-cause deaths, lung cancer, and other pulmonary pathologies [24,25,26]. The relationship between air pollution and viral respiratory infections has been poorly investigated, but a recent study from Murtas and Russo [27] describes a significant association between the increased mortality observed in winter 2016–2017 in Milan (Lombardia) and a combination of influenza and high levels (60 and 70 μg/m^3^) of PM_10_. Furthermore, a specific link between the mortality rate of the 2002 SARS-CoV-1 infection in China and air pollution has been evidenced [28]. Many papers are now appearing describing correlations between air pollutant and SARS-CoV-2 spreading. Different reports from China, which was first affected by the pandemic and has relevant pollution problems, found significant links between different pollutants, including PM_2.5_, and COVID-19 confirmed cases [29,30] and similar associations were evidenced in the United States [31]. In Italy, the association of pollution with increased rates of COVID-19 cases was first proposed by Conticini et al. [32]. Soon after, Fattorini and Regoli [33] demonstrated a statistically significant correlation with various pollutants (NO_2_, O_3_, PM_2.5_ and PM_10_) with the number of COVID-19 cases in 71 Italian provinces. The association with total confirmed and daily new cases of infection and with total deaths was then confirmed in the city of Milan (Lombardia) [34]. During the preparation of the present manuscript, a paper from Frontera et al. reported a correlation between regional level of PM_2.5_ and infection rates [35], also suggesting the up-regulation of ACE-2, driven by pollution, as a candidate molecular mechanism to explain the phenomenon.

Being confirmatory of previous general results, data from our study give an in-deep look at the strength of correlation between PM_2.5_ and the extent of the COVID-19 outbreak. In particular, we performed correlation analysis: (i) considering data from all Italian provinces and (ii) considering the COVID-19 cases and outcomes normalized by the population (incidence and mortality rate) or by the infected population (case fatality risk). This approach allowed us to take into account the high variability in both population density and air pollution levels, which are typical conditions of the Italian territory, and which might represent confounding factors in the correlation analysis. Indeed, we obtained higher correlation coefficients than previously reported data evaluating the absolute number of cases [34]. Furthermore, correlating air quality data collected in a time window moved back a few days compared to the epidemiological data, we ascertained that the level of people’s exposure to the environmental “risk factor” was persistent in the analysed period, and that the exposition period (4–5 days) was enough to induce a biological response in human tissues.

The analysis showed that the infection rate triples for average PM_2.5_ concentration levels increasing from 10 to 25 µg/m^3^ and the case fatality risk doubles for average PM_2.5_ concentration levels increasing from 10 to 22 µg/m^3^, strengthening the supposed role of PM_2.5_ as an enhancer of both SAS-CoV-2 infectivity and virulence, e.g., the severity of the disease as measured by its lethality. The demonstration of a definite implication of air pollution in the present viral pandemic should have strong implications for future directions of environmental policies from national and international governments. However, it is difficult to imagine, in the near future, a firm commitment to reduce pollution from lawyers and economical stakeholders. Thus, it is essential to identify the molecular pathways specifically triggered by prolonged exposition to pollutants, and which may in turn act as predisposing factors for viral infection. This knowledge would allow the design of prophylactic or treatment strategies to mitigate the increased risk to exposed populations. Enhanced persistence of the virus in the air, promotion of a pro-inflammatory state, immune dysregulation, disturbance of pulmonary surfactant homeostasis and increased expression of the viral receptor ACE-2 [36] have been proposed as possible links between air pollution and COVID-19. The latter represents a general mechanism supposed to increase the susceptibility to SARS-CoV-2 infection through an elevated number of viral receptors on the host cells. The novel coronavirus enters the cell through binding of the capsid Spike protein to the cellular surface protein ACE-2, an enzyme involved in regulation of cardiovascular physiology and with a clear role in regulation of inflammation processes. The differential expression level of ACE-2 in various human tissues explains the COVID-19 symptomatology: it is extensively expressed in the upper part of the esophagus, in the lung, in enterocytes [37]. This suggests that pathophysiologic conditions involving increased expression of ACE-2 in the upper respiratory tract and lungs, making available more viral entry sites, might enhance susceptibility to infection and eventually exacerbate the progression of the disease. Accordingly, the different prevalence and severity of COVID-19 in male and females gave rise to the hypothesis of differential, gender-related expression of ACE-2. Even if a clear evidence of such sex difference is not available, an interesting paper from Gemmati et al. [38] recently emphasized that ACE-2 is an X-linked gene, and that mosaicism in heterozygous female may contribute to protective mechanisms compared to the hemizygous male. Similarly, ACE-2 up-regulation following treatment with anti-hypertensive drugs such a angiotensin AT1 receptor blockers, has been proposed as contributing to the increased susceptibility of aged peoples to COVID-19 [39]. On the contrary children, having very a low SARS-CoV-2 infection rate, present a notable reduction of ACE-2 in nasal epithelium compared to adults [40]. Finally, in smokers increased ACE-2 expression has been reported compared to non-smokers [41], suggesting smoke as an additional risk factor for SARS-CoV-2 infection. The issue is debated, since early epidemiological data do not support the hypothesis, albeit important bias in these statistics has been highlighted [42]. However, lung inflammatory conditions other than smoking have been related to dysregulation of ACE-2 expression, such as bacterial infections, chronic obstructive pulmonary disease and pulmonary fibrosis [43,44,45].

The eventual up-regulation of ACE-2 in response to pro-inflammatory stimuli might be explained by its physiological role in the renin-angiotensin system. This pathway includes the angiotensinogen-converting enzyme (ACE) and ACE-2, which act with opposite effects to regulated inflammation: whereas ACE elicits production of pro-inflammatory factors, ACE-2 hinders their expression, enhancing anti-inflammatory molecules. The balance between ACE and ACE-2 activities determines the progression or the quenching of the inflammation process and the exposure to pro-inflammatory molecules stimulates, as a defense mechanism, the expression of ACE-2. Air pollution, particularly PM_2.5_, are among the exogenous triggers of inflammation in upper and lower airways. Indeed, in vivo experiments using a mouse model clearly demonstrated that instillation of PM _2.5_ in the lungs induces ACE-2 over-expression [46], and that following persistent exposure to the noxious particles, the augmented ACE-2 activity induces protection against inflammation.

In summary, the hypothesized scenario is that prolonged exposition to PM_2.5_ of the population in North Italy promotes a pro-inflammatory milieu in the airways, inducing stable up-regulation of ACE-2 as a cellular response aimed to contrast the progression of the inflammatory state. However, this causes the exposition, on the surface of respiratory cells, of a greater number of docking sites for the entry by the virus, favoring infectivity. Notably, following the binding of the viral spike protein, the level of ACE-2 on the cell surface decreases [47], shifting the ACE/ACE-2 balance towards activation of the inflammatory cascade, which represent a hallmark of COVID-19.

Here we report the first supporting evidence for the proposed biological mechanism, describing the presence, in the regulative region of the ACE-2 gene, of putative consensus motifs for the transcription factor AhR. AhR is stimulated by many endogenous and exogenous signals, including environmental pollutants such as PAHs and other noxious molecules contained in PM [48,49,50], and different ligands may specifically activate different genes’ subsets [51]. Besides the primary role in chemical defense by up-regulation of detoxifying xenobiotic enzymes, transforming toxicants into metabolites more easily excreted from the body [52], AhR acts to regulate microbial defense, organ development, immunity and inflammation [53].

The specific subsets of genes regulated by ligand-activated AhR are determined by modulation of its activities through cooperation with a plethora of regulative proteins, [53] and by the specific DNA sequences in the target genes. Indeed, the extent to which a target gene is up-regulated depends on the number, the sequence and the position of consensus binding motifs. These features might affect the affinity of the transcription factor for the DNA sequence and also the eventual cooperation with other regulative proteins bound to different DNA motifs.

We identified a total of nine putative DNA consensus motifs for the binding of AhR (2 XRE, 7 NC-XRE), proximal to various putative binding motifs for core and regulative transcriptional factors (Figure 5). This observation is strongly suggestive and supporting of the hypothesis that pollution-induced over-expression of ACE-2 may favor SARS-CoV-2 infectivity. However, to definitely demonstrate that AhR may control the actual protein level of ACE-2, in vitro studies investigating the effect of disruption (by mutational analysis) of putative AhR binding DNA motifs in the ACE-2 gene should be performed.

## 5. Conclusions

It is to be pointed out that a positive correlation between COVID-19 epidemiologic data and PM_2.5_ concentration levels does not imply a direct and univocal causation, but PM_2.5_ pollution is certainly one of several factors that influenced the pandemic outbreak in Northern Italy in the period February–March 2020. Interestingly, population density seems far less important than PM pollution in COVID-19 diffusion dynamics. Further investigations are needed to confirm that air pollution may modulate ACE-2 expression by activation of the AhR, and comparison of ACE-2 expression in the airways of people living in high polluted areas, compared to people living in healthier environments, would be fundamental.
